# ASSOCIATIONS BETWEEN DAILY STRESSOR CONTROL AND COGNITIVE FUNCTION

**DOI:** 10.1093/geroni/igad104.0366

**Published:** 2023-12-21

**Authors:** Eric Cerino, Brady McClaskey, Yesenia Cruz-Carrillo, David Almeida

**Affiliations:** Northern Arizona University, Flagstaff, Arizona, United States; Northern Arizona University, Flagstaff, Arizona, United States; Northern Arizona University, Flagstaff, Arizona, United States; The Pennsylvania State University, State College, Pennsylvania, United States

## Abstract

Perceived control is an important psychosocial correlate of cognitive health and aging. Most prior research examining this association has focused on global aspects of control, ignoring influences of more dynamic aspects of specific areas of control, such as control over daily stressors. Using data from the third wave of the Midlife in the United States study and the National Study of Daily Experiences (N=992, Mage=67.67, SD=10.34, 57.20% Female), we examined how control over different types of stressors (arguments, avoided arguments, work stress, home stress, network stress) was associated with cognitive performance. Over eight consecutive days, people reported their perceived control over stressors they had experienced. Participants also completed a telephone-based battery of tests measuring executive function (EF) and episodic memory (EM). Hierarchical regression analyses adjusted for number of daily stressors, age, sex, race, and education. The facilitative role of daily stressor control for cognitive health depended on age and the type of stressor experienced. For EF, greater control over arguments was associated with better EF (Est. = 0.07, SE = 0.03, p < .05). For EM, there was a stressor control by age interaction (Est. = 0.01, SE = 0.01, p < .05) such that greater control over home stress was associated with better EM among comparatively older adults (Est. = 0.07, SE = 0.03, p < .05). Results suggest that perceiving control over daily stressful experiences, especially arguments and home stress, may serve as a psychosocial resource for cognitive health in older adulthood.

